# Public Views on Using Mobile Phone Call Detail Records in Health Research: Qualitative Study

**DOI:** 10.2196/11730

**Published:** 2019-01-16

**Authors:** Kerina Helen Jones, Helen Daniels, Sharon Heys, David Vincent Ford

**Affiliations:** 1 Population Data Science School of Medicine Swansea University Swansea United Kingdom

**Keywords:** qualitative research, mobile phone use

## Abstract

**Background:**

Mobile phone call detail records (CDRs) are increasingly being used in health research. The location element in CDRs is used in various health geographic studies, for example, to track population movement and infectious disease transmission. Vast volumes of CDRs are held by multinational organizations, which may make them available for research under various data governance regimes. However, there is an identified lack of public engagement on using CDRs for health research to contribute to an ethically founded framework.

**Objective:**

This study aimed to explore public views on the use of call detail records in health research.

**Methods:**

Views on using CDRs in health research were gained via a series of three public workshops (N=61) informed by a pilot workshop of 25 people. The workshops included an initial questionnaire to gauge participants’ prior views, discussion on health research using CDRs, and a final questionnaire to record workshop outcome views. The resulting data were analyzed for frequencies and emerging themes.

**Results:**

At the outset, most participants (66%, 40/61) knew that location data were collected by operators, but only 3% (2/61) knew they were being used for health research. Initially, the majority of the participants (62%, 38/61) was content for their anonymous CDRs to be used, and this increased (80%, 49/61) after the discussion explained that safeguards were in place. Participants highlighted that terms and conditions should be clearer, as should information to phone users on data collection, privacy safeguards, sharing, and uses in research.

**Conclusions:**

This is the first known study exploring public views of using mobile phone CDRs in health research. It revealed a lack of knowledge among the public on uses of CDRs and indicated that people are generally amenable to the use of anonymized data for research, but they want to be properly informed and safeguarded. We recommend that public views be incorporated into an ethically founded framework for the use of CDRs in health research to promote awareness and social acceptability in data use.

## Introduction

### Background

Mobile phone penetration is constantly rising and is predicted to exceed 5 billion users by 2019; the number of mobile connections already exceeds the world population at over 8 billion [1]. Call detail records (CDRs) are collected passively each time a mobile phone user connects to a mobile network, by either voice call or short message service (SMS) text message. The record generated includes the starting time of the call (or SMS text message), its duration, the caller’s and receiver’s phone numbers, and the locations of the activated towers. Locations can be made more precise via tower triangulation and Wi-Fi connections [2]. Billions of CDRs are collected by mobile network operators (MNOs) such as Orange, O_2_, and EE: they are essential for service operation and are used for billing, monitoring data usage, and targeting customers according to their cell phone use [2]. MNOs may make subsets of CDRs available for research under various data governance regimes [3], enabling the location element in CDRs to be used in a variety of health geographic studies, such as tracking population movement and infectious disease transmission, as shown in a recent review [4].

CDRs are not health data *per se* but can be used alone or in conjunction with other datasets for health research. Just using CDRs alone, researchers have been able to model how to disseminate emergency information during an epidemic in the Ivory Coast [5], work on ways to arrest contagious diseases at an early stage in Belgium [6], and map human mobility after a natural disaster to inform humanitarian response in Nepal [7]. Value is added when CDRs are combined with other datasets for research. In general, these are not linked at the individual level but are overlaid in aggregated form (or at least anonymized) so that individual identities are not exposed. Some examples included in our review are given as illustrations of this kind of study. CDRs with population data and incidence data of Dengue fever were used to predict the timing and spatial extent of disease outbreak in Pakistan [8]. CDRs were used with data on confirmed malaria cases in Namibia to identify areas where malaria surveillance should be increased [9]. CDRs have been used to model likely malaria importation into Zanzibar by combining them with ferry traffic data and malaria surveys [10].

Public perceptions on the use of person-based health and administrative data for research have been, and still are, the subject of extensive work. We define health data as information relating to the health status of individuals, typically, as collected in the course of care provision. We use the term administrative data to include broader public service information such as records on education, housing, and social services. Understanding Patient Data is a prime example of an initiative to support public engagement in the reuse of health data for research [11], along with the Data Saves Lives campaign [12]. In 2014, the Economic and Social Research Council (ESRC) and the Office for National Statistics commissioned a report to explore public understanding and views of administrative data and data linkage [13]. During 7 workshops held with members of the public, it came to light that the public had only limited knowledge of data use in social research, despite acknowledging the importance of data in digital societies. Participants questioned why such research had to take place; their main concerns centered on the risk of reidentification and the level and limits of security protecting the databases where information was held.

The Welcome trust published a report in 2015 on public opinion about the use of health service data for research by commercial companies. Overall, 6 workshops were conducted and researchers found in general that members of the public felt that their identifiable health data should not be shared without their explicit consent and that the risk of reidentification from anonymized datasets was a concern. Participants expressed feelings of mistrust over the use of their health data by commercial companies and questioned the possible motivation for their use [14]. As a view from the opposite perspective, that is, the use of commercial data for research by the public sector, the ESRC commissioned a study in 2015 of public views on private sector data being used for social research. A series of 3 workshops was held, and the findings showed broad support for the reuse of data. The dialogue with the public alleviated many of their concerns about privacy and security and about the role commercial companies can play in research for public benefit [15].

Although there have been surveys of public views on other aspects of mobile phone usage [16], a 2015 seminal work observed that there was no known literature on public perceptions of using CDRs for health research [2]. Despite updated searches, and a review of CDRs in health research [4], no published work on public perceptions of using CDRs for health research was identified. There are studies on public views of using mobile phone apps for monitoring health conditions, but these are distinct and outside our area of interest.

### Objective

Due to the importance of public engagement on the reuse of data for research and the dearth of published work, the aim of this study was to gain public views via a series of workshops and to use the information gained to contribute to an ethically founded framework for the socially acceptable use of CDRs for health research.

## Methods

### Ethical Approval and Study Documentation

Public knowledge on the extent of passive and active data collection via mobile phones, the uses the data are put to, and views on acceptability were gained via a series of 3 public workshops. Ethical approval for research with public participants was obtained from the Swansea University Medical School Research Ethics and Governance Committee. Participants were invited to attend a workshop and did so voluntarily. Information sheets and consent forms were provided, and these set out what participants could expect while taking part in the study and assured them that no identifiable information would be collected, and any comments made would not be attributed to them.

### Pilot Workshop

To inform the structure and content of the public workshops, a pilot workshop was held on March 7, 2017, for members of the Population Data Science department, Swansea University Medical School, and 25 participants attended voluntarily, giving their consent to take part. This department was chosen because it includes experienced data analysts, computer scientists, data managers and data-facing researchers, as well as mobile phone users. It was anticipated that their knowledge would be valuable and insightful in shaping the workshops for more general groups. All the workshops (pilot and public series) were informed by a literature review of studies using mobile phone data for health research [4], and participants were provided with information on examples with their respective benefits and limitations. The types of data that are collected by mobile phone operators and the uses that these data are put to (including health-related research) were introduced. Examples of research studies using CDRs alone and in conjunction with other datasets were given, as described in the introduction. This was intended to enable the participants to consider the types of research that could be conducted, along with the respective risks and benefits.

Participants were introduced to the purpose of the pilot workshop, and to gauge prior knowledge, they were asked for a show of hands in answer to the following questions:

1. Did you know that mobile phone network operators routinely collect data on your location? (The distinction between MNOs and phone handset manufacturers is made for clarity).
2. Did you know that mobile phone network operators and third parties are using the data for research?
3. Have you read the terms and conditions of your mobile phone network operator?

This was followed by a short presentation with examples of research studies using mobile phone CDRs. For example, characterizing the patterns of malaria transmission in Namibia [9], the location of hospitals in relation to travel time following a myocardial infarction or stroke for public health planning in Senegal [17], and monitoring exposure to air pollution in Belgium [18].

Participants were then asked to consider the following in an informal discussion:

1. How aware do you feel about data being collected via mobile phones and likely potential of data for health research?
2. How do you see the pros and cons of using mobile phone data in health research?
3. Any other comments or suggestions regarding workshops with the public?

Finally, the participants were asked to write down their responses to the following:

1. What data do you believe are collected by your phone?
2. How does the operator use the data or make them available to others?
3. What in your view are the data governance issues and risks?
4. How do you feel about these data collection and use?
5. What do you think should be included in the terms and conditions?
6. Which data users or sectors are more acceptable or less acceptable?
7. What do you think a general public group would know?
8. What do you believe the general public would think?

### Public Workshops

The findings of the pilot workshop were used to shape the public workshops. The first of these took place on June 14, 2017, with a convenient workforce group as part of a Swansea University seminar series. Participants were employees of the University from various departments and disciplines; they included academics, researchers, students, and administrators. Overall, 21 people attended the workshop (5 men and 16 women). The second workshop took place on June 28, 2017, with the Consumer Panel for Data Linkage who provide a public perspective on information governance issues in connection with big data and data linkage research in Swansea University–based data initiatives [19]. Overall, 14 people took part in the workshop (7 men and 7 women). The final workshop was held at Pembrokeshire College of Further Education on September 6, 2017, with an adult group attending level 3 health and social care. In total, 26 people (6 men and 20 women) attended the workshop on this occasion. The total number of people who attended the public workshops was 61, and this number is used as the denominator in presenting the results. The age breakdown is shown in Table 1. As the data were collected in age bands, mean age and SD are not shown.

To gauge the representativeness of the sample compared with the UK population, it was compared with the 2011 census figures [20]. The age bands are slightly different in the census, but are close enough to provide an indicative measure. Moreover, we used 18 to 25 years range, as we did not include anyone less than 18 years of age, whereas the census category is 15 to 24 years. The census covers a broader age range than our sample, and so the percentages in the age bands have been adjusted to mirror our range being 100%. Having done this, we have for the census the following: 15 to 24 years: 17%, 25 to 34 years: 18%, 35 to 44 years: 18%, 45 to 54 years: 18%, 55 to 64 years: 17%, and 65 to 74 years: 12%. This indicates that our sample is heavier in the younger (18-45 years) age bands and lighter in the older (46-75 years) age bands.

All the public workshops followed the same format. At the beginning of the workshop, participants were asked to complete a questionnaire (Multimedia Appendix 1) based on their prior knowledge. This questionnaire covered mobile phone use, knowledge of the collection of mobile phone data, their use in research, and the participants’ willingness for their mobile phone data to be used for health research purposes.

**Table 1:** Numbers of public workshop participants in age bands.

Workshop	Age band (years), n (%)						Participants, n (%)
	18-25	26-35	36-45	46-55	56-65	66-75	
1	1 (5)	7 (33)	9 (43)	4 (19)	0 (0)	0 (0)	21 (100)
2	0 (0)	3 (21)	3 (21)	1 (7)	2 (14)	5 (36)	14 (100)
3	14 (52)	8 (31)	3 (12)	0 (0)	1 (4)	0 (0)	26 (100)
All	15 (25)	18 (30)	15 (25)	5 (8)	3 (5)	5 (8)	61 (100)

They were also asked if they had read the terms and conditions of their mobile phone operator, as a show-of-hands. Participants were presented with examples of the following:

- Mobile phone contract terms and conditions highlighting the types of data that are collected and how they are used
- Other types of day-to-day Big Data collection (eg, supermarket loyalty cards)
- Commercial uses of mobile phone data (retail, council planning, and transport models)
- Health research using phone data (as for the pilot workshop)

A general discussion followed using the following questions as prompts:

- What data do you believe are collected by your phone?
- What in your view are the data governance issues and risks?
- How do you feel about this data collection and use?
- What do you think should be included in terms and conditions?
- Which users or sectors are more acceptable or less acceptable?

Further explorations focused on how the public should or could be involved and informed about research using mobile phone data considering the formats in which the data are collected and used, the practicalities of seeking meaningful consent, the acceptability of agreement via MNO terms and conditions, and the implications for individuals and society. Finally, participants were asked to complete a second questionnaire (Multimedia Appendix 2) covering some of the same topics as initial to assess if finding out more via the workshop had altered their opinions on the collection and use of mobile phone data for health research. Questionnaire responses were collected in an anonymous format, but a unique number was applied to each set so that before and after responses for each individual could be compared. Quantitative responses were analyzed as frequencies in IBM SPSS (v.22), and free-text qualitative responses were analyzed thematically by manual assessment and comparison between members of the research team for consensus on theme identification and data convergence.

## Results

### Pilot Workshop

Beginning with the initial 3 show-of-hands questions on prior knowledge, all 25 participants knew that MNOs collect data about their customers, and 24% (6/25) knew that MNOs and third parties are using the data for research, and no one had read their MNO terms and conditions fully, if at all.

The informal discussion based on questions 4 to 6 yielded interesting points. Participants felt reasonably aware of the types of data collected by MNOs. Examples they gave included the location of the user when making phone calls, which mobile apps were on users’ phones, and the data that users had downloaded onto their phones. The general consensus was that there is potential for these type of data to benefit health research, particularly in population health, and participants felt that using their data for this purpose was acceptable as long as their data were anonymized.

However, some concerns were also expressed such as MNOs might sell location data to (potential) employers or to companies such as insurers for profit. Moreover, there was some concern about the risk of disclosure for individuals who live in remote areas. Participants were in consensus in believing that young people would be more likely to be accepting the use of their mobile phone data in health research because of their high usage of mobile phones. Some felt that they would need certain questions answered before being able to decide whether or not they would be happy with this, for example, how secure the identifiable data are before anonymization, whether real-time data are used, and the levels of aggregation applied.

The written responses (questions 7-14) provided the following collated information. Participants listed the data types they believed to be collected by mobile phones as call data: date, time, start or finish, who called or SMS text messaged; data usage; demographic data; location; financial data; online purchasing history; internet search history; app data (eg, about health); and emails (question 7). Participants believed that mobile phone operators may use data to inform advertising strategies or for improving network and data coverage and services. Some also thought that they may make data available by sharing or selling them to third parties such as insurance companies. It was also noted that data would likely be shared with the government if requested (question 8). In terms of data governance issues, the main points were whether data were anonymized and aggregated to a sufficient standard and whether informed consent had occurred for the identifiable data to be collected and used in the first instance. Risks identified included disclosure and data misuse and the increased possibility of reidentification from the use of multiple datasets (question 9).

In total, 11 participants felt happy for their mobile phone data to be used for research purposes as long as they were not identifiable. Several stipulated that they would prefer this to contribute to an improvement for the general population, not just be used for commercial gain. Others wanted to be able to give fully informed consent as way of guaranteeing that they knew exactly what data were being collected, for what purpose, and to be used by whom. Some felt uncomfortable about their data being used in this way and had continuing questions over the identifiability of the data and the corresponding risks of fraud and malicious use. Some participants were concerned that individual-level data (rather than aggregated) could be released to third parties (question 10).

Participants were in agreement that MNO terms and conditions should be written in basic language and be more concise, but also more explicit, and should include information about how, and with whom, the data would be shared. Opting out to certain uses should be available, rather than an *all or nothing* approach (question 11). Participants suggested that use by all sectors could be made possible, but phone users should have the option of opting out of some or all of them. Participants were not in favor of their data being sold to large commercial companies; those that would use their data to improve health were felt to be more acceptable (question 12).

The final 2 questions invited the group to provide their opinions on what the general public would know and think (questions 13 and 14). These questions were asked because the participants were suspected to be more tech-savvy than the public at large by reason of their data-focused work roles, and their views would help in guiding the public workshops. Potential knowledge among the public was hypothesized to be variable and likely to vary with age. Participants believed that a public group would know less than their group because of their experience with big data and analysis. Several participants suggested that members of the public would be less likely to understand what types of data were being collected and may confuse them with data collected via mobile phone apps. It was thought that some people might be alarmed to find out about data being collected via their mobile phones. Participants considered young people to be more familiar with their mobile device and, therefore, more likely to be aware of and accepting the amount of data that is collected.

Even among this group of people working in a data-intensive field of work, no one had engaged with the information provided in the terms and conditions. The group was reasonably aware of the types of data being collected by MNOs and believed the use of CDR data was beneficial, provided that safeguards were in place. A number of concerns were raised, including consent to collect identifiable data in the first place, the effectiveness of anonymization applied, the security of data systems, the potential for data misuse, and the possibility of data being sold to insurers or employers. The discussions and points raised gave us an insight into the types of issues that may arise in the public workshops.

There were some key learning points from the pilot workshop that helped shape the series of public workshops. First, to avoid any confusion, the types of data that are collected by MNOs were described in sufficient detail so that they could be clearly distinguished from mobile app data. Second, to put the topic in context, other types of big data that are collected day-to-day were presented, for example, the data that are collected by supermarkets via their loyalty cards. Finally, it was noted that although pilot participants admitted to not fully reading the terms and conditions of their mobile phone use, all believed that the terms and conditions needed to be changed in some way. Therefore, it was decided that examples of MNO terms and conditions should be presented during the public workshops, so people could give a balanced and informed opinion on whether they thought the current content was sufficient to constitute informed consent.

### Public Workshops

All the public workshops followed the same format, as described above. To gain an understanding of the participants’ level of familiarity with mobile phones, they were asked some questions about their mobile phone use (Multimedia Appendix 1, questions 3-6). We chose to mention smartphones (a smartphone is taken as a more advanced mobile phone that functions as a small personal computer with full internet access, social media connectivity, apps, and games) and *standard* mobile phones in the questionnaire for clarity and to ensure that both types of mobile phones were included but noting that our particular interest was in CDRs, which are collected by all mobile phones. Questions 3 to 6 were not intended for detailed analysis and so a summary is given here. The majority of public participants (92%, 56/61) owned a mobile phone at the time of the workshop (smartphone or standard mobile phone), with the other 5 making use of a family member’s mobile as needed. All but 2 specified that they used a mobile several times a day, 1 used it several times a week, and for the remaining participants, mobile phone use was seldom. All participants said they used a mobile for making phone calls and SMS text messaging and for accessing the internet and emailing. Those with a smartphone noted that they used it for apps, watching videos, playing games, finding places (global positioning system), and several used their phones for reading and making diary appointments. None of the participants had read the terms and conditions.

**Figure 1:**
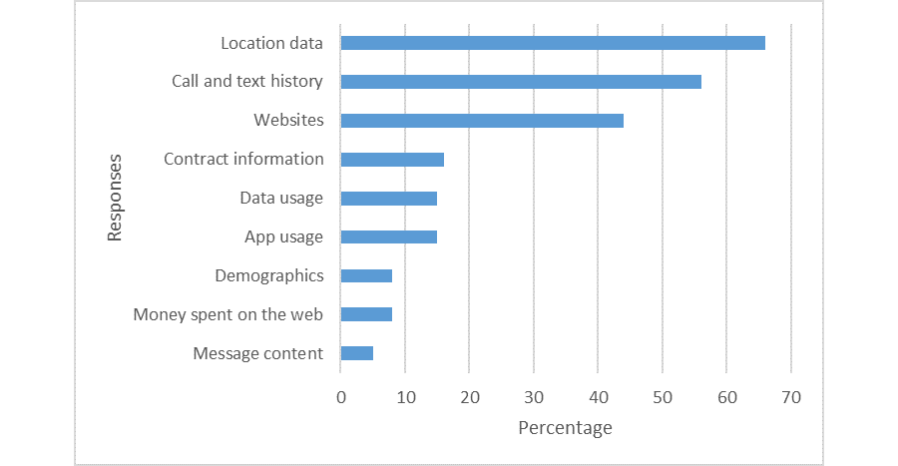
Public views on the types of data collected by mobile network operators in the course of phone use.

On the basis of the responses to questionnaire 1 (Multimedia Appendix 1), at the outset of the workshops, a majority (61%, 37/61) of the participants knew mobile phone operators collect data about the mobile phone user, 11 (18%, 11/61) were not aware of this, and 13 (21%, 13/61) were unsure. When asked to list which types of data they thought that mobile phone operators were collecting (Figure 1), without prompt, the most popular response was mobile phone user location (66%, 40/61).

**Figure 2:**
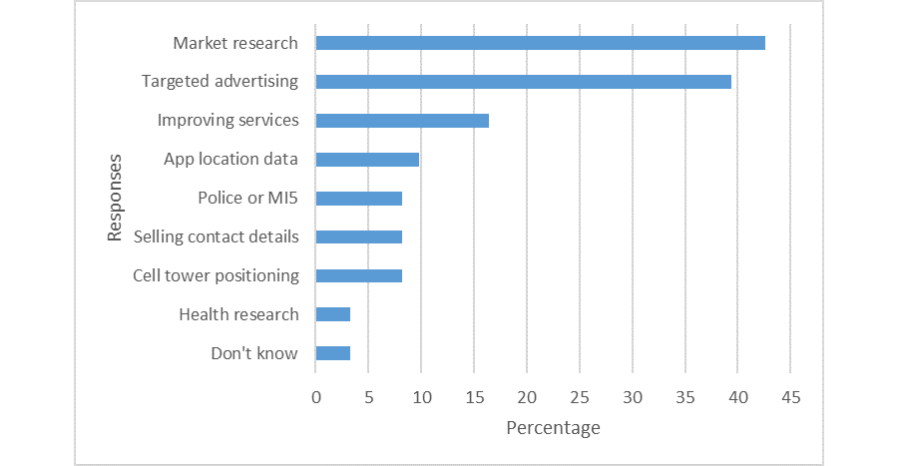
Public responses on how mobile phone data are being used.

In terms of how they believe data are used, a variety of uses were listed in the responses, with market research and targeted advertising being the most frequent (43%, 26/61). However, only 2 (3%, 2/61) participants were already aware that the data were being used in health research (Figure 2).

Despite having little knowledge of health research using mobile phone data, most of the participants (62%, 38/61) agreed that they would be happy for their mobile phone data to be used for this purpose. In total, 4 (7%, 4/61) recorded that they would not be happy for this to occur, and 19 (31%, 19/61) were unsure. Participants were also asked to comment on their response to this question. Overall, 6 people stated that they were happy for their data to be used in this manner as long as their data were safeguarded and anonymized, and 4 participants stated that their data could be used in this manner only if their consent was sought. Other participants explained that they would also be happy with this, on the condition that they were given more information. For example, participants felt that they would want to know what kind of research their data were being used for; one stipulated that they would want to be given the ability to exclude him or her on a project-by-project basis, and another wanted reassurance that the data would not be sold for profit. Overall, 2 participants were keen to be given the opportunity to take part in health research in this way.

Following a presentation of examples and a general discussion (as outlined above), participants were asked to complete a second questionnaire (Multimedia Appendix 2) before leaving the workshop. Having received information regarding mobile phone data and health research, participants were asked again whether they were happy for their data to be used. More participants (80%, 49/61) compared with 62% (38/61) were happy for the data collected via their mobile phone to be used in health research after the workshop than before. 

**Figure 3:**
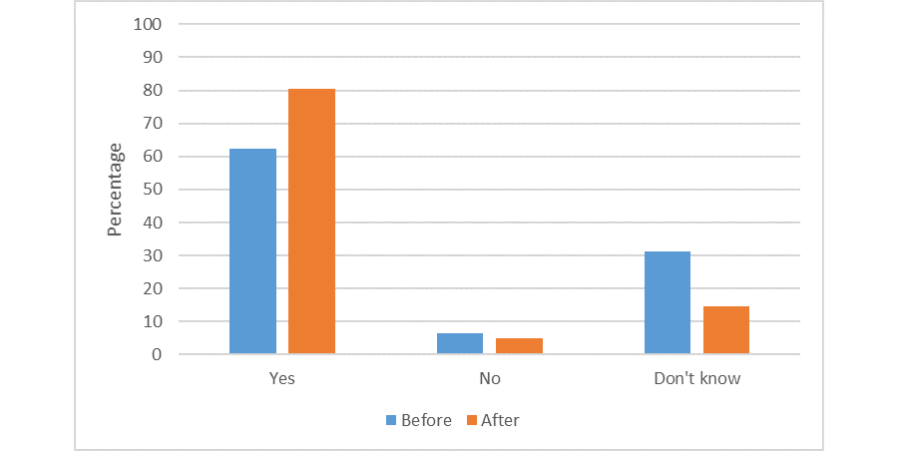
Public views on willingness to use mobile phone data for research before and after the workshop.

Although the majority trend among those who changed their minds was to a more positive viewpoint, there was a small degree of crossover as some became less happy for their data to be used. In total, 13 participants (21%, 13/61) explained that they had changed their minds as they had not been aware that their data was being used for health research in a positive way. Moving in the other direction, 1 participant said they was more concerned as they had been unaware so much data were being collected, and another stated that they felt more concerned but did not give a reason. The comparison between the outset and exit responses is shown in Figure 3.

**Figure 4:**
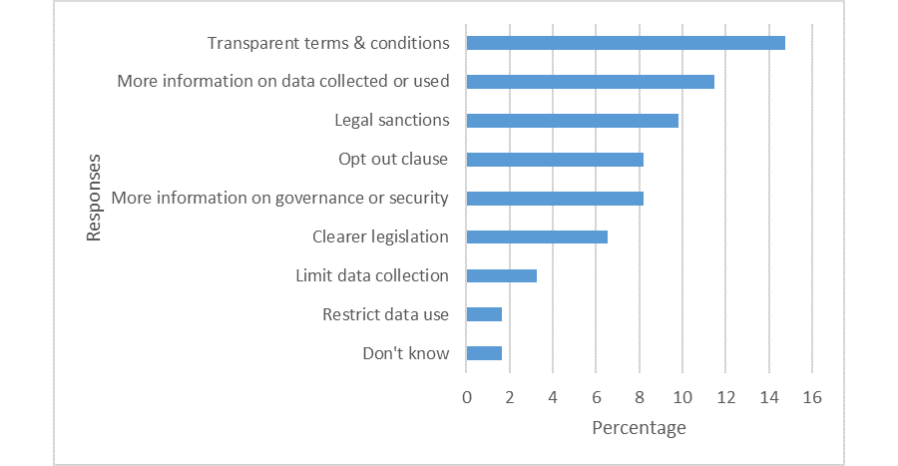
Public views on how to address concerns about mobile phone data use.

The public identified a variety of benefits and concerns in using mobile phone data for health research. Benefits fell into 2 broad categories: (1) improvements in health, for example, *optimize hospital locations*, *public health, track disease spread* and *cures, treatments, and extended health expectancy* and (2) advancements in big data, for example, *easy access to large datasets*, *a cheap and easy method of data collection*, *detailed profiling of a cohort to understand people*, and to *link demographics to health outcomes*. The views about concerns were varied; however, the most frequent was the risk of breaching their anonymity (33%, 20/61), followed by data being sold for commercial gain (25%, 15/61), and unknown third-party use (21%, 13/61). Views on how to address these concerns are illustrated in Figure 4 and include greater transparency, clearer information and options for users, and better data governance.

The majority (84%, 51/61) of participants said that information on the use of anonymized mobile phone data for health research should be included in the terms and conditions. They should be written in simple language to include details on who their data were being shared with, which data were being shared, and why. An opt-out system was also suggested where mobile phone users could choose specifically if and when their data could be shared. The majority was content for MNOs to share their phone data with academia (59%, 36/61), whereas government (34%, 21/61) and charities (26%, 16/61) were less popular options. Only 2 participants were happy to have their data shared with insurance companies and 5 with the pharmaceutical industry. Overall, 6 participants said they would like to be involved with research using mobile phone and health data. They indicated they would have an appetite for influencing topics for research, taking part in further research activities, and advising on public engagement and dissemination strategies.

#### Key Learning Points

At the outset of the public workshops, the majority was aware that a variety of data items were collected routinely, but a sizeable proportion either did not know or were unsure. This suggests that although there is a level of awareness among the general public about data collection by MNOs, there are many with limited knowledge. Of those who were aware, there was a reasonable grasp of the types of data collected, but very few people knew the data were being used for health research. Although, a majority of participants were happy for their mobile phone data to be used for this purpose, quite a proportion was unsure or unhappy. When asked what would make people more comfortable with their data being used for health research, the responses included:

- Data being safeguarded and anonymized
- Consent being sought for usage in research
- More information on types of research
- The option to opt-in and out on a project-by-project basis
- Reassurance that the data would not be sold for profit

At the end of the workshops, a greater number of participants stated they were happy for their phone data to be used for health research than at the outset, with the principal reason for the changed viewpoint being that they had not realized the data could be put to beneficial health uses. Participants listed some notable benefits of using phone data in this way but also some important concerns, notably:

- Risk of reidentification
- Data being sold for commercial gain
- Unknown third-party use

A number of suggestions on how to improve the terms and conditions were made by the workshop participants and these were:

- Use of more basic language
- Wording to be more concise but more explicit
- Inclusion of information about how, and with whom, data would be shared
- Allowing opt out to different data sharing options, rather than an *all or nothing* approach

- Stronger, more transparent, information governance

Participants were also in favor of information about the use of anonymized mobile phone data for health research to be included in the terms and conditions. Although the majority was happy for their data to be shared with academic institutions, few people thought it acceptable for data to be shared with pharma and insurance companies. In summary, the public workshops indicated that people want clearer and more information; to be informed with whom and for what purpose data are shared; their views to be taken into account; more assurance of good data governance; and greater transparency and overt accountability from the MNO.

## Discussion

### Principal Findings

This is the first known study to explore public views on the use of mobile phone data, specifically CDRs, for health research. The workshops revealed many relevant and potentially valuable findings.

### Knowledge and Engagement

As anticipated, the pilot group members were more knowledgeable than the general public groups at the outset. All members of the pilot group knew that MNOs collect data about phone users, compared with less than two-thirds among the general groups. Similarly, there was considerably greater awareness of data being used for research. This supported the value of running the pilot group with data-focused staff and of their insights to inform the public workshops. None of the total participants (25+61) said they had read the terms and conditions.

### Concerns

The main concerns raised across the groups were in relation to data privacy and perceived inappropriate use. These centered on the nature of consent to collect identifiable data, the risk of reidentification in data purported to be anonymized, the potential for unknown use and misuse, and data being sold to potentially discriminatory parties.

### Solutions

The solutions suggested by the groups largely converged on being provided with more information, more choice, and greater assurance of proper data governance. Participants wanted to know that their data were safeguarded and wanted reassurance that their data would not be sold for profit without their engagement. They wanted to know about the types of research that might take place, by whom and for what purpose, with public benefit being an important factor. Depending on the form of data being used, participants wanted to be asked for informed consent or to be able to opt-out of certain data uses. Across the groups, participants wanted clearer and more transparent terms and conditions.

An interesting and somewhat ironic finding was that having clearer terms and conditions and more information on data collection and use were strongly recommended although no one read the information already provided. This is an apparent contradiction and one that raises questions as to why, if people have concerns and want to know more, they do not read the information given to them. This is a common problem, not limited to mobile phone contracts, and one for which there have been a number of social experiments. The complexity of the wording used in various social media terms and conditions was recently highlighted as requiring a university degree to understand them fully [21]. Another study concerned a group of over 500 students signing up to a fictitious social media channel. None of the students read the terms and conditions well enough to notice that they had agreed to hand over their first-born child [22]. A further example involved over 7500 people agreeing to forgo the rights to their immortal soul by failing to read the terms and conditions for a gaming site download [23]. This phenomenon is also observed in other domains. In a survey completed by 550 direct-to-consumer genetic testing customers, most respondents considered themselves aware of privacy issues and the risk of troubling repercussions of data donation to be negligible. However, over 50% men and almost 30% women also said they had not read the terms and conditions [24].

These examples, as well as the situation with mobile phone terms and conditions, leave us with doubts about the adequacy of informatory processes in some spheres. However, they also highlight the dilemma of where the responsibility lies, as to whether the onus should be more on the individual or the data collector [25]. Clearly, it is important that people read terms and conditions, but there are issues with the current formats of these documents that discourage them from doing so. It is possible that this is because of requiring to agree to the terms and conditions to obtain the phone and also the user-unfriendliness of the layout, with extensive small print in often opaque language. As it appears that the current terms and conditions are not hitting the spot, we propose that the format of information, and the way it is provided to the public, needs to be revised.

The public engagement workshops were particularly revealing about people’s awareness and viewpoints on the use of mobile phone CDR data. Although MNOs are clearly profit-making organizations, participants did not want their data used for profit. This could be considered to be a naïve position as commercial gain via provision of a service is the raison d’être of MNOs, or perhaps it is because of the separation in our perceptions between the use of our phones and the systems that operate behind the scenes [26]. It should be acknowledged that there is sometimes a lack of understanding among the general public about identity disclosure risks in the use of anonymized or strongly pseudonymized data and aggregated data. Similarly, there may be some misunderstandings about the regulatory and legal requirements of using such data. However, the concerns raised are valid, as although something is lawful, it might not be socially acceptable [27]. Some argue that we have entered a state of surveillance realism characterized by a combination of unease and resignation to the use of our data [28]; however, this does not negate the need for more meaningful engagement with the public and the onus on us all to engage with our social responsibilities [25].

In common with a study on other private sector data being used for social research, dialogue with the public alleviated many of their concerns and clarified the role commercial companies can play in research for public benefit [15]. Furthermore, it had already been identified that there is an absence of a clear, holistic, ethical, and regulatory framework to guide research using CDRs [2]. The findings of this study support the need for such a framework, incorporating public views to guide its development.

### Limitations

The main limitations of this study were the number of participants and the time available for the workshops (1 hour each). A longer time might have allowed more deliberative engagement and drawn out additional points. Although the findings of the workshops cannot claim to be fully representative of the public at large, as they are weighted toward younger or middle-aged adults, they should provide food for thought for MNOs and other parties with an interest in using mobile phone CDRs for research. It is possible that bias was introduced in drawing out the themes, but we aimed to avoid this by coming together to discuss data reduction and approach consensus.

### Conclusions

Mobile phone CDRs are increasingly being used for health research with a geographical element. This novel study engaged with a cross-section of the public to gain their perspectives on the use of mobile phone data for health research. It was evident that this is a topic that has lacked public engagement in the past, and it showed that while the public recognizes the value of CDRs for health research, important concerns were raised and solutions suggested. The findings of this study lead us to recommend future work on gathering the views of a wider spectrum of participants to verify our findings, ascertain why people do not engage with the information provided in the terms and conditions and what a better information vehicle would look like, and further explore perceptions around profit-making from mobile phone and other networked device data. Crucially, our findings support the inclusion of public views into the development of an ethically founded framework for the socially acceptable use of CDRs in health research. We suggest that the design of this study might be of value to others seeking to work with the public on this important topic and in relation to data from other networked devices.

## Appendix

Multimedia Appendix 1 Questionnaire 1: Public workshop prior knowledge questionnaire.

Multimedia Appendix 2 Questionnaire 2: Public workshop exit questionnaire.

## Abbreviations

CDRs: call detail records
ESRC: Economic and Social Research Council
MNO: mobile network operators
SMS: short message service
