# Supplementary material for: Public Views on Using Mobile Phone Call Detail Records in Health Research: Qualitative Study
Source: JMIR Mhealth Uhealth. 2019 Jan 16;7(1):e11730. doi: 10.2196/11730 (PMC6352010; doi:10.2196/11730)
Supplement: Multimedia Appendix 1 [file mhealth_v7i1e11730_app1.pdf]

Appendix 1

Questionnaire 1: Public workshop prior knowledge questionnaire[1]

Please answer the following questions to provide information about your mobile phone use and your views on the use of mobile phone network data.

Section A. Brief information about yourself

1. Please indicate your age range from the options below:

18 to 25 years

26 to 35 years

36 to 45 years

46 to 55 years

56 to 65 years

66 to 75 years

Over 75 years

2. Are you?

Male

Female

Other

Prefer not to say

Section B. Information about your mobile phone use

Compared to a standard mobile phone, a smartphone is defined here as a more advanced mobile phone that functions as a small personal computer with internet access, social media connectivity (Facebook, Twitter, etc.), general applications and personalized applications (such as games, fitness monitoring, etc.).

3. Do you use a smartphone?

Yes

No

4. If yes, how often do you use a mobile phone?

Several times a day

Once a day

---

1 Questionnaire spacing has been minimised for publication.

Several times a week

Once a week

Seldom

5. What do you use a mobile phone for? Please tick all that apply

Making phone calls

Texting

Accessing the internet

Emailing

Using apps

Playing games

Reading

Watching videos

Finding places when travelling/GPS

Diary appointments

Other uses? (Please state)

6. If you do not use a smartphone, what are the reasons for this? Please tick all that apply

Happy using a standard mobile phone

Don’t need a smartphone

Smartphones are too expensive

Smartphones are too complex

No interest in using a smartphone

Don’t need a smartphone or a mobile phone

Other reasons? (Please state)

Section C. Your views on the use of mobile phone network data

Mobile phone network operators collect data via mobile phones about the phone owner and the use of the phone.

7. Did you know that data are collected about the mobile phone user and how the mobile phone is being used?

Yes

No

Not sure

8. What types of data do you believe the mobile phone operator collects via mobile phones? Please list up to 6 types

9. How do you think the data collected via mobile phones are used? Please list up to 6 uses

10. Are you happy for data collected via your mobile phone use to be used in health research?

Yes

No

Don’t know

Any comments on this?

Thank you for completing questionnaire 1.
