# Supplementary material for: Public Views on Using Mobile Phone Call Detail Records in Health Research: Qualitative Study
Source: JMIR Mhealth Uhealth. 2019 Jan 16;7(1):e11730. doi: 10.2196/11730 (PMC6352010; doi:10.2196/11730)
Supplement: Multimedia Appendix 2 [file mhealth_v7i1e11730_app2.pdf]

Appendix 2

Questionnaire 2: Public workshop exit questionnaire

1. Having attended the workshop today, are you happy for data collected via your mobile phones use to be used in health research?

Yes

No

Don’t know

2. If you have changed your viewpoint compared to before the workshop, please state what made you change your mind

3. Please list up to 3 benefits and 3 concerns of using mobile phone data in health research

Benefits	Concerns

4. What could be done to address your concerns? (Please list up to 3 possible solutions)

5. Do you think that information about the use of anonymized mobile phone data for health research should be included in the terms of service?

   Yes
   No
   Don’t know

6. With whom would you be happy for mobile phone operators to share your mobile phone data? (e.g. academic institutions, charity, pharmacological/private companies, insurance companies, government).

7. What information would you like mobile phone operators to provide you with regard to the data they are sharing?

8. How would you like to be involved with research using mobile phone and health data?

Thank you for completing questionnaire 2.
